# School-based sexuality Education in Europe and Central Asia

**DOI:** 10.1093/eurpub/ckac129.740

**Published:** 2022-10-25

**Authors:** J Marquardt

**Affiliations:** Federal Centre for Health Education, Berlin, Germany

## Abstract

**Introduction:**

In 2016, the BZgA and the European Network of the International Planned Parenthood Federation initiated an extensive survey regarding the development and current status of sexuality education (SE) in Europe and Central Asia, which included 25 selected countries of the European WHO Region. Since 2000, remarkable progress has been made in the region in developing and integrating SE curricula in school settings. The majority of the surveyed countries teach basic elements of SE in schools. Yet, in about half of the countries, there is still some reticence in understanding the benefits of SE for the health and well-being of young people. In countries with fully developed comprehensive SE programmes, the school is the main source of information on sexuality for young people. In the other countries young people tend to rely on information from friends or peers and the internet. The findings also showed a gap in teacher training on sexuality education.

**Methods:**

The Survey included a detailed questionnaire, among member associations of IPPF EN and government agencies responsible for SE. A random sample was drawn from the 50 states of the region which is considered representative for the entire region. All 25 IPPF EN members and 16 of the 25 government agencies responded.

**Results and discussion:**

Remarkable progress has been made in developing and integrating SE in formal school curricula. In 11 of the 21 countries, SE is a mandatory teaching subject, and in six additional countries it is partly mandatory. In four additional countries it is optional. Despite the progress, there are still shortcomings and gaps in providing the full spectrum of SE.

**Conclusions:**

If provided, SE tends to focus primarily on the biological aspects and prevention of HIV/STIs and unwanted pregnancy. There is a need to broaden the spectrum of topics that are addressed to include gender equality, sexuality, violence and sexual abuse, human rights and empowerment.

